# Supplementary material for: The Creation of True Two-Dimensional Silicon Carbide
Source: Nanomaterials (Basel). 2021 Jul 10;11(7):1799. doi: 10.3390/nano11071799 (PMC8308388; doi:10.3390/nano11071799)
Supplement: Supplementary file 1 [file nanomaterials-11-01799-s001.zip › nanomaterials-1297101-supplementary.pdf]

nanomaterials

MDPI

Supplementary Materials

The Creation of True Two-Dimensional Silicon Carbide

Sakineh Chabi [1,*], Zeynel Guler [1], Adrian J. Brearley [2], Angelica D. Benavidez [3] and Ting Shan Luk [4]

[1] Department of Mechanical Engineering, University of New Mexico, Albuquerque, NM 87131, USA; zeynelguler@unm.edu
[2] Department of Earth and Planetary Sciences, University of New Mexico, Albuquerque, NM 87131, USA; brearley@unm.edu
[3] Department of Chemical and Biological Engineering and Center for MicroengineeredMaterials, University of New Mexico, Albuquerque, NM 87131, USA; asanch18@unm.edu
[4] Center for Integrated Nanotechnologies, Sandia National Laboratories, Albuquerque, NM 87123, USA; tsluk@sandia.gov
* Correspondence: schabi@unm.edu

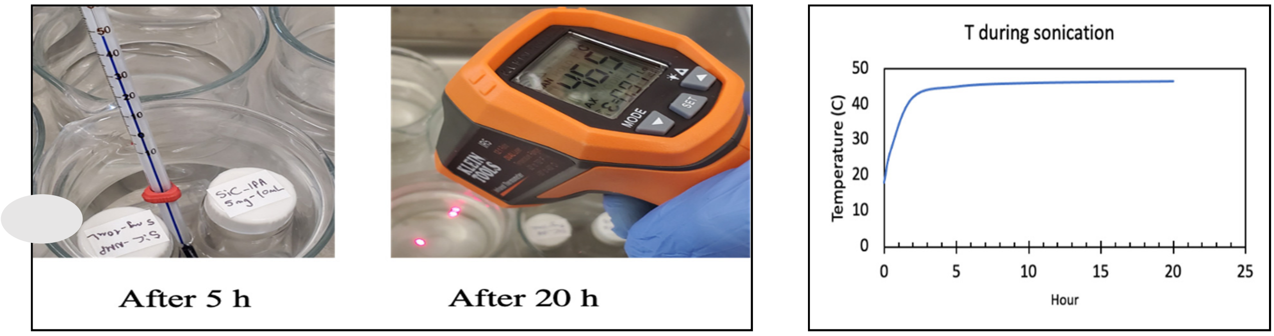

Figure S1. (a) Photographs from the SiC-containing vials during the bath sonication process. As shown, during the exfoliation process, the bath temperature was kept below 50 °C **(b).**

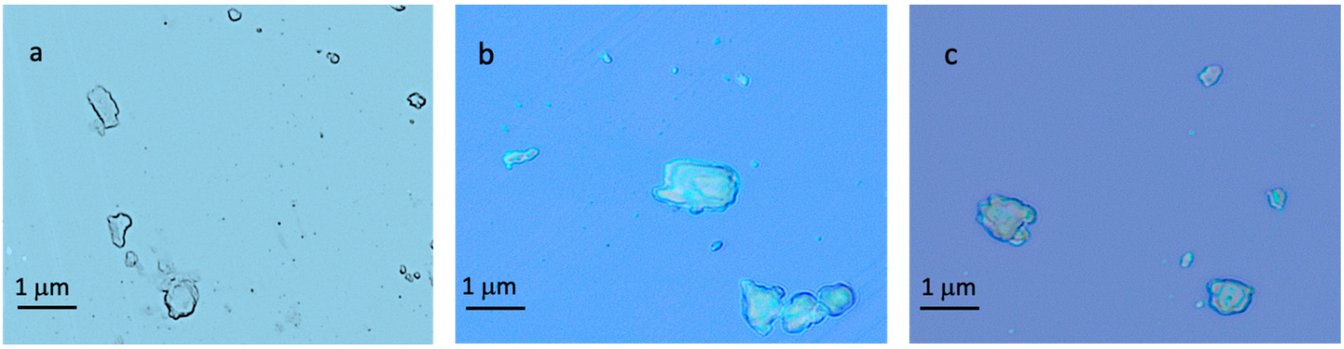

Figure S2. Optical microscopy images of the exfoliated SiC nanoflakes. **(a)**: on glass slide, and **(b,c)** on SiO_2/Si substrates.

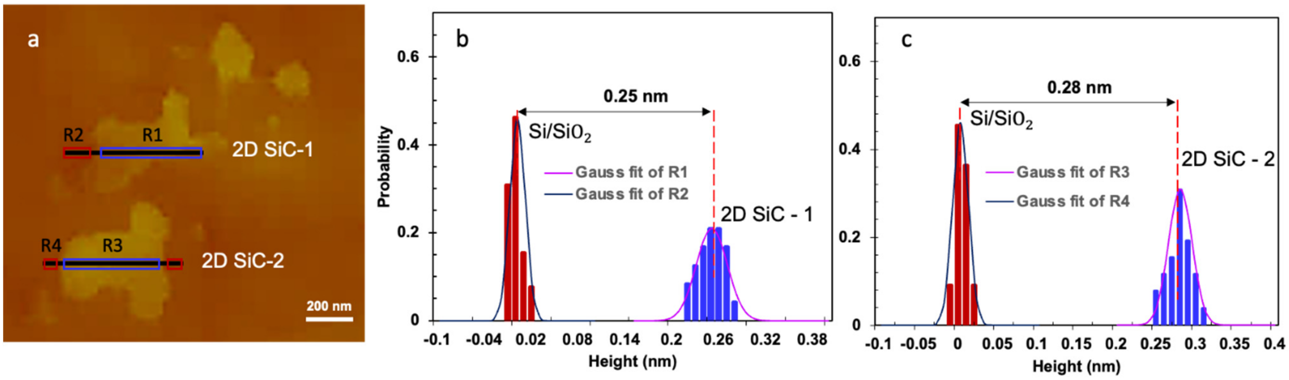

Figure S3. (a)AFM image of the exfoliated SiC nanosheets, and **(b,c)**Height histograms acquired from 2D SiC nanosheets

(SiC-1, and SiC- 2), and SiO_2 substrate. The heigh separation between the peaks of the 2D SiC samples, and silicon wafer are 0.25 (SiC-1) and 0.28 nm (SiC-2), which is consistent with our AFM measurements, further confirming the monolayer nature of the presented nanosheets.

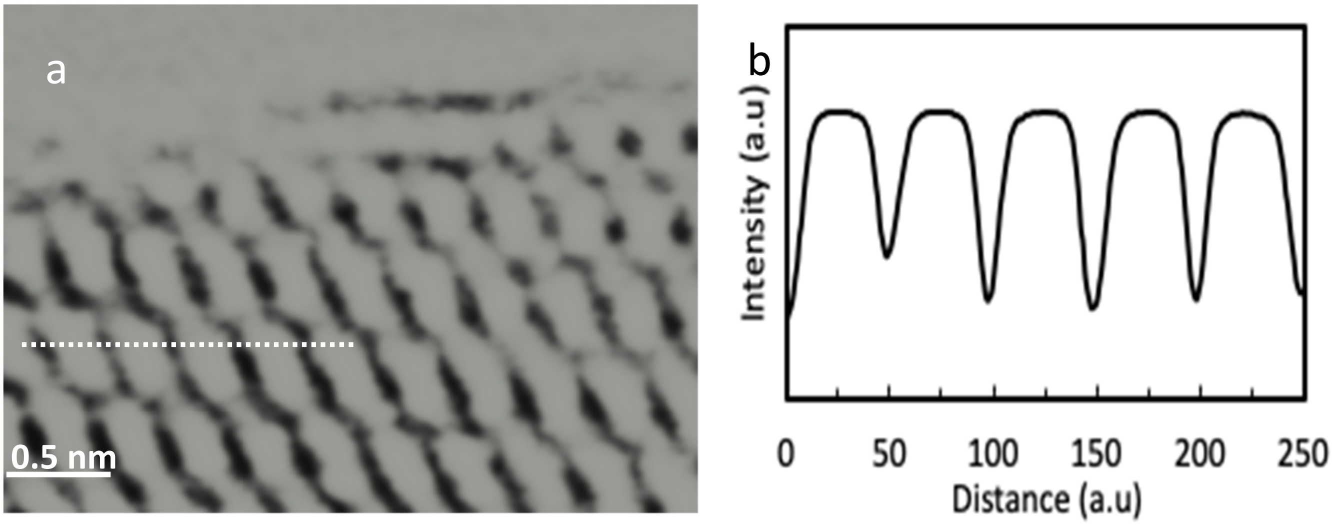

Figure S4. (a) TEM imager from a 2D SiC sample, and **(b)** relative intensity profile along the dot line in (a).

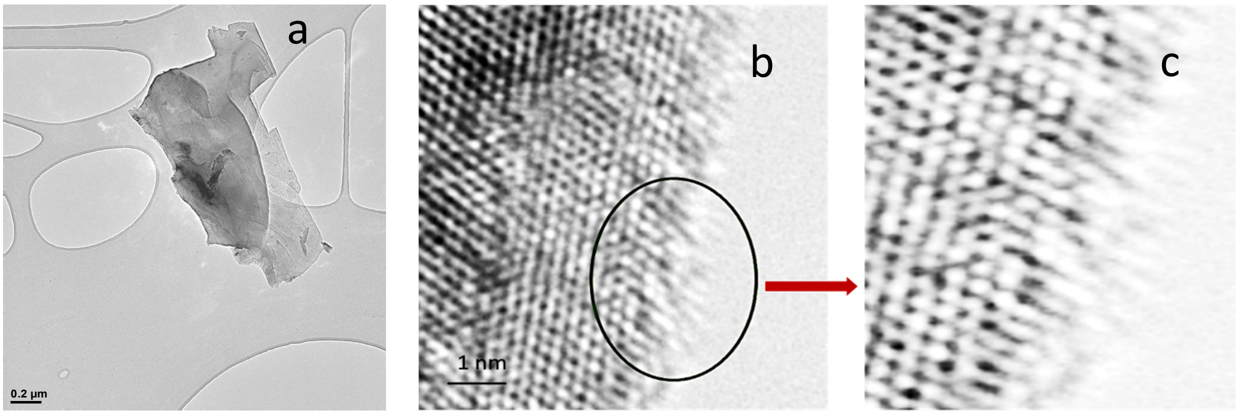

Figure S5. (a) low magnification TEM image of the exfoliated SiC nanosheets. **(b,c)** HRTEM images from another 2D SiC sample. The HRTEM image from the edges of 2D SiC clearly confirms high crystallinity nature of the created nanosheets.

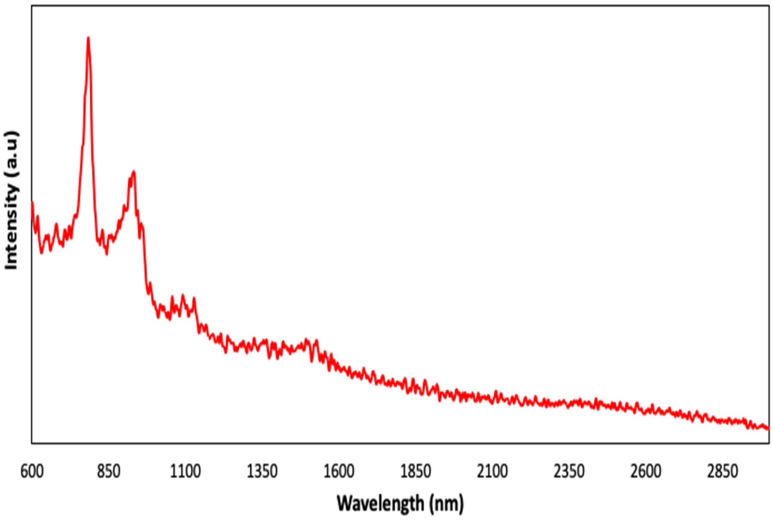

Figure. S6. Raman spectrum from a 2D SiC sample.

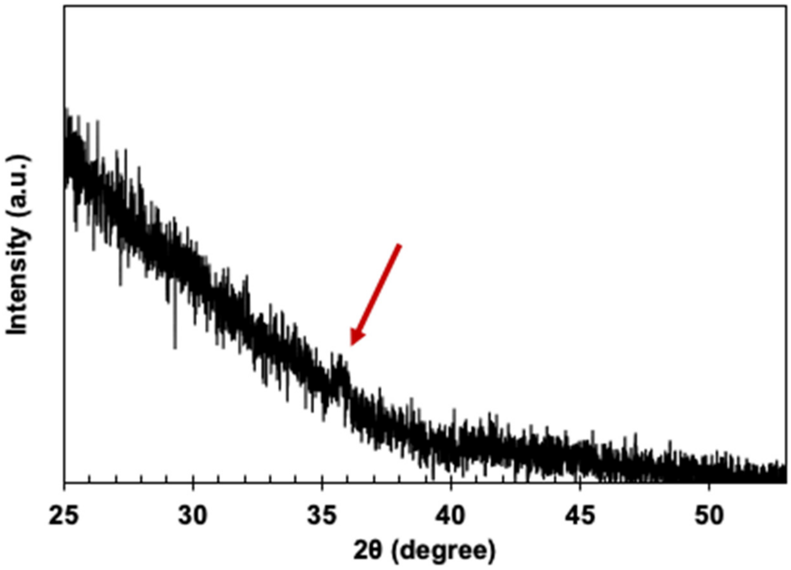

Figure S7. XRD pattern from 2D SiC dispersions. This pattern was acquired after placing more than 50 drops, on top of each other, on a glass substrate. No peaks were observed, when only few drops were placed on the substrate.
